# Theranostic Approaches for Gastric Cancer: An Overview of In Vitro and In Vivo Investigations

**DOI:** 10.3390/cancers16193323

**Published:** 2024-09-28

**Authors:** Ghazal Basirinia, Muhammad Ali, Albert Comelli, Alessandro Sperandeo, Sebastiano Piana, Pierpaolo Alongi, Costanza Longo, Domenico Di Raimondo, Antonino Tuttolomondo, Viviana Benfante

**Affiliations:** 1 Department of Health Promotion, Mother and Child Care, Internal Medicine and Medical Specialties, Molecular and Clinical Medicine, University of Palermo, 90127 Palermo, Italy; domenico.diraimondo@unipa.it (D.D.R.); bruno.tuttolomondo@unipa.it (A.T.); 2 Ri.MED Foundation, Via Bandiera 11, 90133 Palermo, Italy; gbasirinia@fondazionerimed.com (G.B.); amuhammad@fondazionerimed.com (M.A.); 3 NBFC—National Biodiversity Future Center, 90133 Palermo, Italy; 4 Pharmaceutical Factory, La Maddalena S.P.A., Via San Lorenzo Colli, 312/d, 90146 Palermo, Italy; sperandeo@lamaddalenanet.it (A.S.); piana.sebastiano@lamaddalenanet.it (S.P.); 5 Nuclear Medicine Unit, A.R.N.A.S. Civico Di Cristina e Benfratelli Hospitals, P.zza N. Leotta 4, 90127 Palermo, Italy; pierpaolo.alongi@arnascivico.it (P.A.); costanza.longo@arnascivico.it (C.L.); 6 Advanced Diagnostic Imaging-INNOVA Project, Department of Radiological Sciences, A.R.N.A.S. Civico Di Cristina e Benfratelli Hospitals, P.zza N. Leotta 4, 90127 Palermo, Italy

**Keywords:** gastric cancer, targeted therapy, in vitro, in vivo, targeting sites, imaging, theranostics

## Abstract

**Simple Summary:**

Gastric cancer (GC) represents a major global health challenge, ranking as the second leading cause of cancer-related deaths. The high mortality rate is primarily due to late-stage diagnoses, which limit effective treatment options. Recent research emphasizes the development of targeted therapies and radiation immunotherapy as promising alternatives to traditional approaches. The review authors summarize recent advancements in targeted therapies for gastric cancer, focusing on both laboratory and clinical studies. They explore targeted approaches that have shown potential for improving diagnostic accuracy and treatment effectiveness. The findings of this paper may significantly impact the research community by highlighting the importance of personalized medicine in gastric cancer treatment. By identifying specific molecular targets and utilizing nanotechnology for drug delivery, these advancements aim to enhance treatment efficacy while minimizing systemic toxicity. Overall, the review suggests the potential for improved prognosis and treatment strategies through targeted therapies and precision medicine.

**Abstract:**

Gastric cancer (GC) is the second most common cause of cancer-related death worldwide and a serious public health concern. This high death rate is mostly caused by late-stage diagnoses, which lead to poor treatment outcomes. Radiation immunotherapy and targeted therapies are becoming increasingly popular in GC treatment, in addition to surgery and systemic chemotherapy. In this review, we have focused on both in vitro and in vivo research, which presents a summary of recent developments in targeted therapies for gastric cancer. We explore targeted therapy approaches, including integrin receptors, HER2, Claudin 18, and glutathione-responsive systems. For instance, therapies targeting the integrin receptors such as the αvβ3 and αvβ5 integrins have shown promise in enhancing diagnostic precision and treatment efficacy. Furthermore, nanotechnology provides novel approaches to targeted drug delivery and imaging. These include glutathione-responsive nanoplatforms and cyclic RGD peptide-conjugated nanoparticles. These novel strategies seek to reduce systemic toxicity while increasing specificity and efficacy. To sum up, the review addresses the significance of personalized medicine and advancements in gastric cancer-targeted therapies. It explores potential methods for enhancing gastric cancer prognosis and treatment in the future.

## 1. Introduction

Globally, gastric cancer (GC) ranks second in malignancy-related deaths. For this malignancy, five-year survival is lower than 30%, depending on geographical regions. Detecting gastric cancer at an advanced stage leads to a high mortality rate [1]. Surgery and chemotherapy [2] are the primary therapies used to treat gastric cancer, and radiotherapy [3,4,5], immunotherapy [6], and targeted therapies are also being explored. However, GC survival rates are inadequate. Early detection improves gastric cancer prognosis and treatment efficacy [7]. The World Health Organization classifies gastric cancer into four types: papillary, tubular, mucinous, and poorly cohesive. Clinical effectiveness is inconsistent due to no clear biological classification system [8,9]. Adenocarcinomas of the stomach mucosa are the most common type. Others can cause stomach, muscle and lymphoid cancers [10].

### 1.1. Molecular Pathology in Gastric Cancer

**Figure 1:**
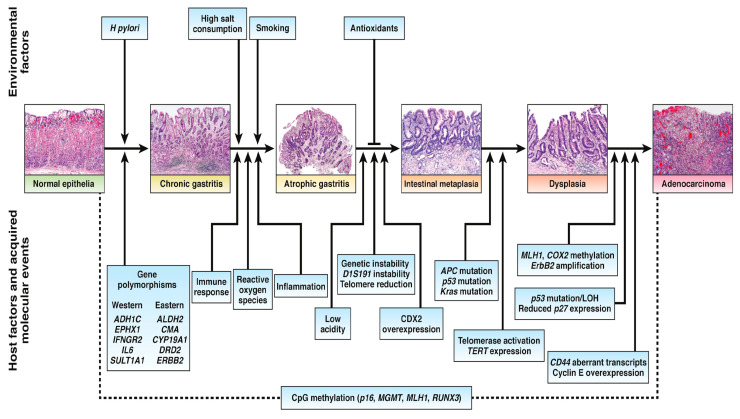
Important steps in intestinal-type gastric cancer (GC) pathogenesis that showed molecular and environmental factors. Reprinted from Ref. [12]. Copyright 2015, Gastroenterology. This article is an open-access article distributed under the terms and conditions of the Creative Commons Attribution (CC BY-NC-ND 4.0) license.

Based on the Lauren classification, there are two main types of gastric adenocarcinoma: diffuse and intestinal. Inflammation of Helicobacter pylori can cause gastric cancer. It has a role in metaplasia and atrophy of the gastrointestinal anatomical district. Pangastritis without atrophy is the cause of diffuse gastric cancer [11]. GC pathogenesis consists of chronic atrophic gastritis and intestinal metaplasia (IM) (Figure 1) [12]. Family background, diet, alcohol consumption, smoking, and Helicobacter pylori infection all affect GC development [13]. Studies appear to implicate N-nitroso compounds in GC [14]. There was a strong correlation between GC development and alcohol consumption in patients carrying polymorphisms in aldehyde dehydrogenase 2 (ALDH2) and the ALDH2*1/*2 genotype [15]. GC detects different molecular biomarkers differently. GC is affected by several factors, such as apoptosis-regulating factors, cell cycle regulators, markers that alter membrane properties, multidrug-resistant proteins, and unstable microsatellites. In positive GC cases, overexpression and amplification of human epidermal growth factor receptor 2 (HER2) ranged from 6% to 30% [16]. Mouse double minute 2 homolog (MDM2) expression level was significantly higher in gastric cancer and intestinal metaplasia than in chronic gastritis [17]. Retinoblastoma protein (pRb) and cyclin D1 are more abundant in the early stages of GC [18]. Overexpression of Mucin proteins like MUC1, MUC2, MUC5AC, MUC6, and GC indicates that Mucin inhibitory function plays a crucial role [19]. Multidrug resistance-associated protein 2 (MRP2) predicts chemotherapy response. Initial chemotherapy treatments for tumors fail to work because of increased MRP2 expression [20].

### 1.2. The Role of Targeted Therapy in the Treatment of Advanced Gastric Cancer

As immuno-oncology and cancer pathophysiology have improved, cancer diagnosis and treatment methods have evolved [21,22,23,24]. Immune checkpoint inhibitors (ICIs) aim to improve advanced GC treatment. The other approach to treating advanced gastric cancer is adoptive cell therapy, antibodies against vascular endothelial growth factor A (VEGFA), cancer vaccines, and chimeric antigen receptor (CAR) T-cell therapy [25,26]. Some GC biomarkers can serve as pathogenesis indicators. Non-invasive biomarker identification for early diagnosis and prognosis of GC has unexpected results. Liquid biopsy and serum biomarkers are both clinical biomarkers for GC. Serum biomarkers such as CEA, CA19-9, AFP, CA72-4, and CA12-5 have limited ability as a detection marker for early gastric cancer [27]. Carcinoembryonic antigen (CEA) and carbohydrate antigen (CA19-9) are common markers used in clinics, with CEA rising in later gastric cancer stages and CA19-9 elevated in pancreatic cancer [28]. AFP-positive gastric cancer usually has advanced stages [29]. Further research is needed to confirm CA72-4 and CA125 as early gastric cancer markers [30]. Cell-free DNA (cfDNA) and circulating tumor DNA (ctDNA) are viable forms of early diagnosis but are not reliable enough to detect precancerous lesions. CfDNA levels in precancerous tissues are similar to healthy controls, limiting their use [31]. Early diagnosis of cancer requires identifying patients with precancerous lesions at high risk. Cancer-predictive markers include CDH17, TP53 mutations, and KLK10 for precancerous stomach lesions [32,33].

**Figure 2:**
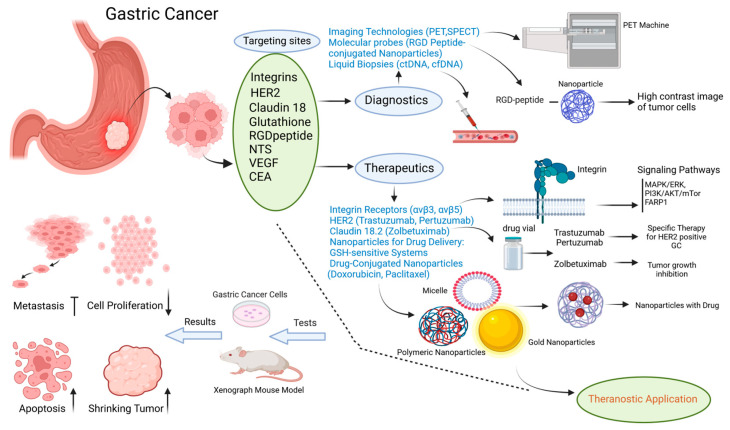
Therapeutic strategies in gastric cancer. A summary of the role of targeting sites such as integrin receptors, HER2, Claudin 18, RGD peptide, glutathione, NTS, VEGF, and CEA has been presented in this figure. All of these strategies are combined with imaging technologies and drug-conjugated nanoparticles for theranostic applications. Moreover, both in vitro and in vivo experiments revealed their impact on signaling pathways as well as their specific effects on tumor growth, apoptosis, metastasis inhibition, and cell proliferation. Note that: ↑ means “increase” and ↓ means “decrease”.

A new form of precision medicine, theranostics, combines diagnostics and therapy [34]. Current and forthcoming perspectives examine theranostic nano-medicine [35] to achieve more precise personalized medicine customized for each patient [36,37,38]. This revolutionary idea encourages us to explore more about nanoparticles (NPs) [39], which are man-made materials ranging in size from tens to hundreds of nanometers. Due to their effectiveness and less harm, they have become increasingly popular in recent years. Theranostic nanoparticles will continue to make advances in clinical oncology as demand for less invasive and more precisely targeted therapies grows. In addition to further experiments and protocols utilizing theranostic approaches to gastrointestinal (GI) tumors, radiolabeled somatostatin analogs are also effective in the diagnosis and treatment of pancreatic neuroendocrine tumors. In routine clinical practice, only two molecular mutations have been validated as predictive therapeutic targets for GI cancers. In advanced gastric cancer, Her2neu is a favorable prognostic indicator, while KRAS is negative [40]. Aside from the overexpression of Her2neu, MET and FGFR2 are considered promising therapeutic targets in gastric cancer due to their oncogenic role [41,42]. To efficiently transport drugs to gastric cancer cells, recent drug delivery strategies have increasingly employed nanotechnology [43]. Figure 2 summarizes a typical experimental workflow investigating theranostic approaches, especially those based on nanosystems (e.g., nanoparticles).

This review aims to elucidate all the known state-of-the-art biomarkers used in in vitro and in vivo experiments. These biomarkers have been considered for future theranostic applications in various GC forms.

## 2. Gastric Cancer Targeting Sites

The discovery of biomarkers in GC makes research evolve toward the optimization of new strategies that are performing better for theranostic applications. Biomarkers, in particular, membrane receptors, are very promising for the creation of tumor-specific receptor carriers suitable for use as diagnostic tracers, for example, in PET and SPECT imaging. In particular, in the field of radiotheranostics, there is an increasing effort to use these biomarkers and to enrich in vitro and in vivo tests using the same macromolecules for dual purposes, i.e., diagnosis and therapy. Furthermore, in theranostics, other strategies, also innovative, concern the use of nanosystems, such as nanoparticles with the ability to convey a chemotherapeutic or a therapeutic radionuclide or other types of ligands to the tumor site. Considering the severity of the pathology of GC and the difficulty of developing personalized medicine protocols for patients affected by this neoplasm, today, it is imperative that the knowledge of the state of the art be exploited globally, beginning with the various targets, to maximize what is already known that Table 1 summarizes the present biomarkers for GC.

The targeting sites of GC are discussed in the following studies.

### 2.1. Integrin Receptor

Integrins are a type of receptor found on cell surfaces that participate in cellular interactions with the extracellular matrix. Among them, the αvβ3 integrin was detected at a high level on the surface of various cancer cells, including gastric cancer. It was also found on activated endothelial cells in tumor blood vessels, while it is barely present in normal cells [69]. Consequently, the arginine glycine–aspartic acid (RGD) peptide, which targets αvβ3 integrin, offers advantages like easy synthesis, small size, high stability, and low immunogenicity, and is commonly used in nanoparticle design for targeting gastric cancer [70]. RGD-coated nanoparticles successfully reached and performed at the site of tumor growth that exhibited a high level of mitochondrial apoptosis and significant antitumor effects with low toxicity both in vitro and in vivo. This approach may improve clinical efficacy while minimizing the side effects of GC treatment [71]. Cheng et al. created an RGD-indocyanine green (RGD-ICG) molecular probe along with a surgical navigation system in mice. This nanoprobe notably boosted both the sensitivity and specificity of their gastric cancer diagnostic method, achieving an accuracy rate of 93.93%. When used during surgery, this probe resulted in a smaller measured tumor diameter (1.8 mm) and reduced operative time by 3.26 times compared to traditional treatment. These results highlight the dual benefits of the RGD-ICG probe in enhancing diagnostic precision and improving gastric cancer surgery effectiveness making promising therapeutic strategies for future applications [44]. In another study reported by Miyamoto et al., integrin α5 could be used as a therapeutic target for preventing the spread of a diffuse type of gastric carcinoma (DGC). Their focus was on identifying the interaction between DGC cells and cancer-associated fibroblasts (CAFs) that contribute to aggressive DGC spread. They conducted the research via in vitro and in vivo tests. For in vitro experiments, they used these human cancer cell lines (NCI-N87, A549, IM95, MKN74, GCIY, 44As3, 58As9). They assessed the invasive capacity of DGC cells when CAFs are present and showed that blocking of integrin α5 or its ligand signaling decreased this invasion. For experiments on the mouse xenograft model, they tested whether the enhancement of monoclonal antibody or elimination of integrin α5 in DGC cells impacted peritoneal distribution. Thus, these experiments suggested that integrin α5 is important for making the interaction between DGC cells and CAFs, boosting the peritoneal distribution and targeting the integrin α5 could be a promising therapeutic strategy for inhibiting the spread of DGC [45].

**Figure 3:**
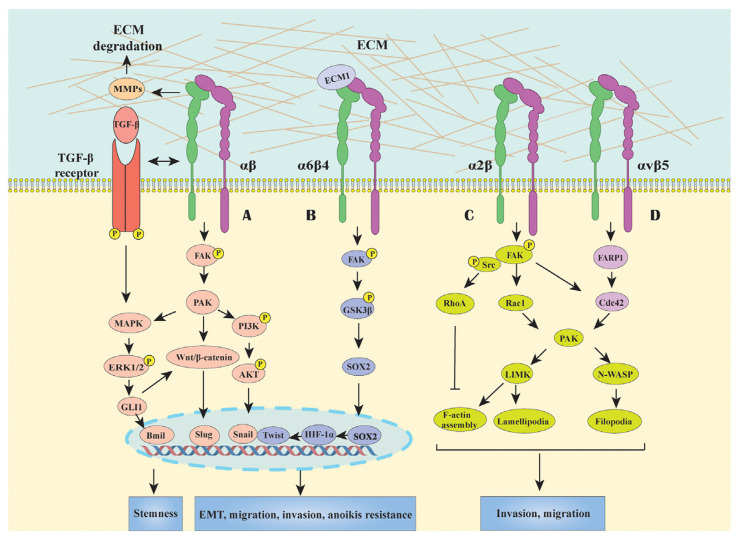
In GC, integrin regulates signal transduction pathways. The majority of integrins start signaling pathways produced by FAK, such as MAPK/ERK, PI3K/AKT, and Wnt/β-catenin, to engage in processes related to invasion, migration, and EMT, as well as to develop stemness and resistance to anoikis, programmable cell death form. Also, by boosting the activity of the downstream molecule Cdc42, the interaction between integrin αvβ5 and FARP1 can promote cell motility. Reprinted from Ref. [73]. Copyright 2021, Frontiers in Molecular Biosciences. This article is an open-access article distributed under the terms and conditions of the Creative Commons Attribution (CC BY-NC-ND 4.0) license.

In addition, Hirano et al. conducted a study and discovered the potential features of the integrin αvβ5-FARP1-CDC42 axis for the migration and invasion of gastric cancer cells and also evaluated the expression level of pleckstrin domain protein 1 (FARP1) as a prognosis marker for gastric cancer patients. The experiments on cells performed on human gastric cancer cell lines like MKN7, MKN45, MKN74, and GSU demonstrated the up level of FARP1 expression increased motility and the structure of cells to a specific form (filopodia) via CDC42 activation when the expression level decreased the motility of gastric cancer cells changed and could be low. In this way, FARP1 promotes cancer cell spread by interacting with the integrin β5 and activating another protein called CDC42. Further, FARP1 levels in patients are linked to cancer spreading. Lastly, blocking FARP1 could be a promising gastric cancer treatment [46]. Zhang and colleagues investigated the integrin-subunit alpha 11 (ITGA11) expression level and its relation to PI3K/AKT signaling in gastric cancer. The in vitro tests performed on GC cell lines (MKN45, AGS) showed knockdown. ITGA11 reduced cell proliferation, invasion, and migration but enhanced apoptosis. In addition, these changes affected the inactivation of the PI3K/AKT pathway. ITGA11 knockdown decreased tumor growth in animal models. Thus, ITGA11 facilitates GC progression through the inflection of the PI3K/AKT pathway. The reader is referred to [72] for an in-depth overview of targeting PI3K/Akt signaling for cancer therapy. These results could show potential for GC prognosis and therapy [47]. Integrins mediate GC signal transduction pathways, as shown in Figure 3 [73]. Integrin-binding ligands labeled with several radioisotopes for theranostic applications in PET imaging are contained in [74,75,76].

### 2.2. Human Epidermal Growth Factor Receptor 2 (HER2)

**Figure 4:**
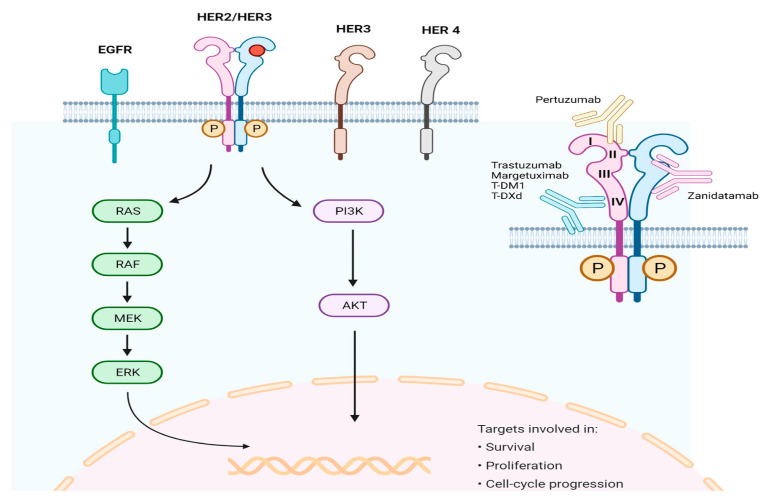
Signaling pathway and antigen-binding region for targeting HER2. Reprinted from Ref. [77]. Copyright 2023, MDPI. This article is an open-access article distributed under the terms and conditions of the Creative Commons Attribution (CC BY-NC-ND 4.0) license.

The human epidermal growth factor receptor 2 (HER2), also called ERBB2, is one of the receptors linked to various processes of tumor cells, like growth, cell death, adhesion, movement, and differentiation (Figure 4) [77]. Much evidence suggests that HER2 is a significant biomarker and a main cause of tumor formation in gastric cancer. Research on GC has found that around 7–34% of tumors showed overexpression in HER2 [78].

Espelin et al. discovered that nanoparticles targeting HER2 in two ways, using trastuzumab and liposomal-encapsulated doxorubicin (called MM-302), showed strong combined targeting and antitumor effects in breast and gastric cancer. By targeting different parts of HER2 receptors, MM-302 and trastuzumab could attach to tumor cells that overexpress HER2 at the same time. This innovative treatment that efficiently targets HER2 overexpressing cells through multiple methods is being considered for a randomized phase II clinical trial, which creates an opportunity for the treatment of HER2-positive cancer patients [48]. However, Castagnoli et al. conducted a study that revealed the reason for the nonresponding to trastuzumab as a gold-standard treatment in advanced HER2-positive gastric cancer (GC). Thus, they assessed the expression level of fatty acid synthase (FASN) and their function in a preclinical model of HER2+ GC and evaluated the inhibition of FASN impacting on resistance to anti-HER2 treatment in HER2+ GC, especially when targeting HER2+ cancer stem cells (CSCs). With the utilization of WB and FACS and also by qRT-PCR and GEP analyses for evaluating the FASN protein and transcript expression level in both sensitive-resistant trastuzumab HER2+ GC cell lines under 2D and 3D culture conditions and found the FASN expression level was higher in 3D cultures, which was in collaboration with high CSC characteristics and worse prognosis. The in vitro and in vivo experiments used a drug (TVB3166) for FASN inhibition that showed a reduction in cancer stem cells and tumor growth in both tests. Therefore, combining this FASN inhibitor with trastuzumab proved more effective than trastuzumab alone. This suggests that targeting both HER2 and FASN could enhance therapeutic approaches for patients who did not respond to trastuzumab [49].

Lu177-radiolabeled pertuzumab has been shown to be effective in theranostic applications targeting HER 2 receptors, which makes this knowledge even more valuable for the development of new PET radiopharmaceuticals that exploit not only binding to these receptors but also internalization and pathways that support their function. Both in vitro and in vivo experiments revealed that the radiolabeled immunoconjugates preserved the synergistic binding of pertuzumab and trastuzumab to HER2 receptors. The present limitations of current radioimmunotherapy treatments may be partially addressed by this dual-epitope targeting strategy to improve outcomes [50].

### 2.3. Claudin 18

Claudins (CLDNs) are situated at the cell membrane in the apical region and have a role in multiprotein junction complexes that regulate the polarity of cells, cell migration, and remodeling of the matrix through the paracellular route, as well as in the proliferation of cells [79]. Thus, enhanced levels of metastatic tumor cell infiltration and migration might be associated with the lack of CLDN expression. However, overexpression of CLDNs has been observed in several cancers, including GC, so it could have a potential for higher metastasis [80]. CLDN18 has been identified as a target for therapy and intensive research in gastric cancer. This is due to its typical expression in normal gastrointestinal cells and stable expression in gastric tumor cells [81].

Ungureanu et al. showed that specific binding of zolbetuximab to the first extracellular domain of CLDN18 splice variant 2 (CLDN18.2) with high affinity can induce antibody-dependent cellular cytotoxicity (ADCC) and complement-dependent cytotoxicity leading to the destruction of gastric cancer cells that express CLDN18.2. The results of this study showed a low prevalence—34.2% of 2055 patients expressing CLDN 18.2, but they suggested the key to novel therapeutic approaches. These findings imply that a revised threshold for CLDN18.2 positivity ought to be taken into account, and computer-based analysis could improve precision in subsequent research [51]. Nishibata et al. reported a study that evaluated the efficacy of zolbetuximab in CLDN 18.2-positive gastric cancer cells in vitro and in vivo. For the in vitro tests, they used NUGC-4, KATO-III, and CLS-103 GC cell lines that showed zolbetuximab killed cancer cells effectively through antibody-dependent cellular cytotoxicity (ADCC) and complement-dependent cytotoxicity (CDC) mechanisms. In vivo tests on syngeneic and xenograft GC mice models assessed the antitumor effects of zolbetuximab as a monotherapy and combined with chemotherapy. Results demonstrated that zolbetuximab significantly inhibited tumor growth in both therapies, particularly when combined with chemotherapy, which increased the infiltration of CD8+ T cells and was more efficient in tumor growth inhibition when coupled with anti-mouse programmed cell death-1 (anti-mPD-1) therapy. Consequently, all the results demonstrated that zolbetuximab was a novel option for both gastric cancer and gastroesophageal junction (GEJ) therapy [52].

Furthermore, targeting CLDN18.2 is a promising immunoPET theranostic strategy for diffuse gastric cancer [82].

### 2.4. Glutathione

Glutathione (GSH) is a natural substance that breaks disulfide bonds. This is a crucial feature because it helps moderate cell growth and keeps chemical components balanced in the body. GSH levels are different inside and outside cells, with lower levels inside. This difference has led to the development of nanoplatforms that react to GSH. These platforms offer a potential way of drug delivery into gastric tumors while minimizing damage to healthy tissue [83]. Shi and colleagues created a special compound by adding paclitaxel (PTX) to polyethylene glycol (PEG) with RGD attached to achieve this purpose. These special (PEG–PTX) combinations form micelles, which break down in low GSH and release PTX in slightly acidic conditions inside cells. Experiments showed that this release in response to GSH effectively blocked tumor growth with minimal side effects. The effectiveness of RGD@Micelles in terms of tumor targeting and tumor inhibition was verified through the use of a mouse xenograft model for gastric tumors. Polymer-drug conjugate micelles show promise as drug delivery nano-vehicles for tumor therapy, as evidenced by their significant therapeutic effects on gastric tumors [53]. Zhang and others proposed an efficient system using a gold/platinum star-shaped core (Au/Pt stars) that targets tumors with GSH-sensitive bounds. In mice, the probe accurately targeted tumors, released therapeutic molecules, such as IR780 for phototherapy, and utilized glucose oxide to generate toxic particles for tumor damage. This system works for GSH but also supports real-time imaging [84,85]. New molecular targets and strategies offer promise for personalized therapy [54].

Xu et al. assessed the expression level of glutathione peroxidase-2 (GPx2) and the correlation of that with clinical factors and prognosis. In vitro tests were performed on different gastric cancer cell lines like MKN-28 GC cells, NUGC-4 GC cells, and MKN-45 GC cells, and human gastric epithelial cell line (GES-1) and showed that (GPx2) knockdown inhibited GC proliferation, migration, and epithelial–mesenchymal transition (EMT) in both in vitro and in vivo tests. For in vivo experiments, they used gastric xenograft tumors and peritoneal metastasis mice models to determine the expression of GPx2 and conducted the procedure of GC proliferation and metastasis. The results revealed that GPx2 expression impacted kynurenines (KYNU)-mediated metabolism, in addition to the involvement of the reactive oxygen species (ROS)-mediated KYNU-kyn-AhR signaling pathway in gastric cancer progression and metastasis following GPx2 knockdown. Finally, the accumulation of ROS due to GPx2 knockdown via the KYNU kyn-AhR regulatory pathway could suppress GC progression and metastasis, which provided evidence supporting the role of GPx2 in GC progression and recognized it as a therapeutic target and novel potential prognostic marker for GC [55].

Another study by Chen et al. examined the impact of GPX7 expression, regulation, and molecular functions on gastric cancer cell lines and human tissue samples. For this aim, they used 2D and 3D in vitro tests performed on various gastric cancer cell lines (AGS, MKN28, MKN45, MKN74, SNU1, SNU5, SNU16). They detected downregulation of GPX7 in all seven gastric cancer cell lines as well as in approximately half of the human gastric cancer samples. This was compared to normal tissues. The methylation analysis of cell lines and samples showed a significant difference compared with normal samples. Additionally, they treated the AGS and SNU1 cells with 5-Aza-2′-deoxycytidine to confirm that GPX7 was silenced by an epigenetic molecular mechanism. A reconstruction of GPX7 inhibited the growth of GC cells in 2D and 3D cell cultures, leading to a suppression of cell proliferation and cell death. Tumorigenesis and gastric cancer progression may be influenced by GPX7 gene inactivation [56].

As an intracellular antioxidant and redox balance regulator, glutathione (GSH) plays a crucial role. Nanomedicines mediated by GSH have been developed as a cost-effective means of diagnosing and treating cancer, including GSH-responsive delivery systems of drugs, GSH-mediated dynamic therapies, immunotherapy, and GSH-triggered cancer imaging and theranostics [86].

### 2.5. Cyclic RGD Peptide

There has been continuous discovery of a sequence that is highly effective with strong attraction for use in biomedical applications. This sequence is among different RGD patterns, including cRGDyk, a cyclic RGD peptide. Mao and colleagues developed nanoparticles composed of silk fibroin carrying a substance called chlorin e6 and having a special tag called cRGDyk for targeting cancer cells. These particles were used to deliver drugs like 5-fluorouracil (5-FU) and conduct photodynamic therapy for gastric cancer treatment. This nanoparticle specifically targeted tumor cells and slowly released drugs while also being effective in photodynamic therapy. When tested in mice with MGC 803 cancer cells, the nanoparticle significantly reduced tumor size while demonstrating excellent biocompatibility and safety. Thus, SF-based NPs are potentially effective drug delivery vehicles, and when combined with photodynamic therapy (PDT) and multimodality therapy, they may be useful as a viable treatment option for cancer in the future [57].

Ding et al. conducted a study that the PEGylated liposome encapsulated ICG, coupled with RGD peptide, as a new system called (RGD-PLS-ICG), which utilizes integrin-mediated pathways for targeted delivery. Through amide binding, RGD is linked to a specific lipid, DSPE-PEG2000-NH2, to achieve this targeting. A thorough examination was conducted to evaluate the properties and stability of these liposomes. In vitro experiments involved selecting SGC7901 cells with heightened integrin α5β1 expression via various lab techniques like polymerase chain reaction (PCR) and Western blotting. To validate the targeting efficacy toward gastric cancer, encapsulated coumarin-6 as a fluorescent substance to observe liposome targeting under microscopy and flow cytometry. For the in vivo study, the investigations involved evaluating the biodistribution of RGD-PLS-ICG using an in vivo imaging system in tumor-bearing mice. Results indicated that RGD-PLS-ICG was more stable and had better UV absorption compared to free-ICG. Confocal microscopy and flow cytometry experiments showed an efficient association of RGD-PLS-encapsulated coumarin-6 with SGC7901 cells, while interactions with other groups were limited. Furthermore, in vivo imaging illustrated an increased accumulation of RGD-PLS-ICG in tumor tissues compared to liposomes without RGD. These findings suggest that RGD-PLS-ICG could be an effective fluorescent dye delivery system specifically for gastric cancer cells overexpressing integrin [58]. 

Wang and colleagues designed RGD-conjugated silica-coated gold nanorods to develop an effective strategy for NIR absorption on the surface of multiwalled carbon nanotubes (MWNTs) by enhancing their covalent interaction with carboxyl groups from in vitro to in vivo tests in gastric cancer cells. For the in vitro test, they used a gastric cancer cell line (MGC803) and human gastric mucous (GES-1) as control cells. By using a simple strategy, they were able to attach covalently silica-coated gold nanorods (sGNRs) to the surface of multiwalled carbon nanotubes. Furthermore, the combination of carboxyl groups on the MWNTs and the amino group on the surface of sGNRs was changed with a silane coupling agent, which made a connection between them. Also, they investigated RGD-conjugated sGNR/MWNT probes and their viability effects on MGC803 and GES-1 cells. For in vivo tests, they prepared gastric cancer-bearing nude mice models by injecting the RGD-conjugated sGNR/MWNT probes via tail and then using an optoacoustic imaging system. In vitro test results showed overexpression of RGD on the MGC803 cell surface; otherwise, expression on the surface of GES-1 was not observed through dark-field microscopy. For this reason, gastric cancer MGC803 cells could have been targeted with the prepared RGD-GNR-MWNT nanoprobes. In vivo outcomes displayed that RGD-conjugated MWNT/sGNR nanoprobes had good biocompatibility and targeted tumor vessels effectively, which enhanced the PA imaging of these vessels. It shows significant potential for photoacoustic imaging and photothermal therapy in the future [59].

Based on this biomarker and compared with RGD monospecific tracers, the newly synthesized heterodimeric PET radiotracer [68Ga]Ga-FAPI-RGD showed improved tumor uptake, prolonged tumor retention, tumor targeting efficiency, and effective pharmacokinetics in mouse pancreatic ductal adenocarcinoma cells that suggested high efficiency in diagnosis and potential for therapeutic uses [60].

### 2.6. Neurotensin Receptors

**Figure 5:**
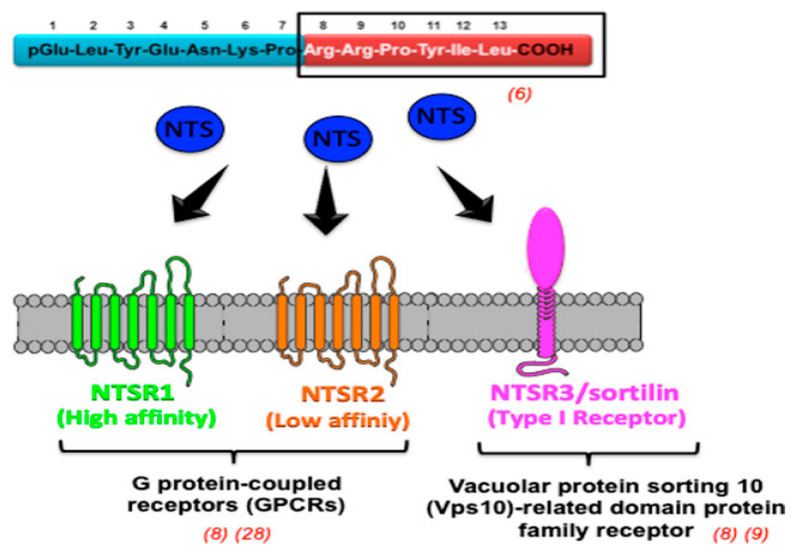
Three remarkable NT receptors, NTR1, NTR2, and NTR3, link to the C-terminal part of 8–13 amino acids of NT. Reprinted from Ref. [89]. Copyright 2020, Cell Death and Disease. This article is an open-access article distributed under the terms and conditions of the Creative Commons Attribution (CC BY 4.0) license.

Neurotensin (NTS) is secreted in the intestinal mucosa through endocrine cells and metabolized by the liver. It functions as a neurotransmitter/modulator in the central nervous system and as a hormone in the peripheral nervous system [87,88]. In the gastrointestinal tract, NTS has a variety of functions. These include promoting mucosal proliferation, controlling gut motility based on muscle type and location, and suppressing gastric acid secretion. By binding to two G protein-coupled receptors, NTSR1 and NTSR2, and a third receptor, NTSR3/sortilin, which is a member of the Vps10-related domain protein family, it promotes the growth of cells in digestive organs such as the stomach, colon, pancreas, and small bowel mucosa (Figure 5) [89].

NT/NTR1 has recently been reported to be a prognostic indicator for head and neck cancer [90]. MAPK/ERK signaling pathways play a key role in regulating cellular motility through the coordination of matrix metalloproteinase (MMP) activity in gastric cancer. Disassembling focal adhesions induced by EGFR impacts cell adhesion processes and controls cell migration and invasion.

Akter. H and colleagues conducted an investigation into the relationship between neurotensin (NT) and matrix metalloproteinase-9 (MMP-9) in gastric cancer. In vitro tests on two gastric cancer cell lines (MKN-1 and MKN-45) and normal epithelial cells (HFE-145) using commercial ELISA kits, fluorescence resonance energy transfer (FRET) assay and Western blotting demonstrated that NT increased MMP-9 activity, cell migration, and invasion in gastric cancer cells, but not in normal cells. It was demonstrated that the protein kinase C (PKC), extracellular signal-regulated kinase (ERK), and phosphatidylinositol 3-kinase (PI3K) pathways played a role. Additionally, SR48692 inhibited NT-mediated MMP-9 activity, invasion, and migration. Thus, NTR1 may be a therapeutic target for gastric cancer [61]. The other experiments were performed on 60 gastric cancer tissue samples and various cancer cell lines (lung, breast, pancreatic, and colon). In vitro tests demonstrated that NTSR1 mRNA levels in gastric cancer tissues and cell lines were higher than those in non-cancerous tissues and other cancer cell lines. Treating with neurotensin (NT) enhanced matrix metalloproteinase-9 (MMP-9) activity and expression, which was reduced by blocking NTSR1. Furthermore, depleting NTSR1 through the Erk signaling pathway decreased the invasion and migration of gastric cancer cells by confirmation of NT-induced metastasis through observing changes in epithelial–mesenchymal transition markers. Therefore, this finding suggests that NTSR1 could be a promising therapeutic target for blocking the invasion and spread of gastric cancer [62].

From a theranostic perspective, for tumor scanning and, more recently, for peptide receptor radiotherapy treatment of tumors, radiolabeled peptides such as neurotensin, substance P, gastrin-releasing peptide, glucagon-like peptide 1, and neuropeptide Y (NPY) have been studied for targeting breast, prostate, ovarian, pancreatic, and brain cancers [91].

### 2.7. Angiogenesis

Angiogenesis is recognized as a highlight feature of cancer, and GC has been identified as a therapeutic target like many other cancers. The family of VEGF, which consists of three receptors, VEGFR1-3, and six growth factors, VEGFA-E, plays a key role in tumor angiogenesis regulation and is often targeted by different anticancer compounds [92]. PI3K plays an important role in the proliferation and survival of several types of cancer cells, including gastric cancer. This signaling cascade facilitates tumor progression in GC by inhibiting apoptosis, including drug resistance, promoting metastasis, and increasing angiogenesis [93]. A change in the PI3K/AKT/mTOR pathway is crucial in GC and other solid tumors for HER2-targeted therapy and chemotherapy resistance [94]. Chen H et al. reported a study that proved to block p-ACT/p-mTOR in gastrointestinal tumor cells in vitro, resulting in specific effects on the AKT/mTOR/VEGF-C/VEGF-D signaling pathway. In vitro testing was conducted using SGC-7901 gastric cancer cell lines treated with p-Akt and p-mTOR inhibitors to determine the relationship between the Akt/mTOR pathway and VEGF-C/-D. Following that, they measured the inhibition rate of cell growth with an MTT assay and utilized a Western blot for protein expression analysis. Also, an ex vivo test was performed using tissue samples from 55 GC patients. Thus, gastric cancer lymphangiogenesis may be associated with the Akt/mTOR-VEGF-C/VEGF-D axis [63].

Long et al. synthesized a pH-responsive liposome (Liposome-PEO, LP) loaded with apatinib (AP) and cinobufagin (CS-1) and coated it with a hybrid membrane (R/C) to improve the bioavailability and reduce side effects of drug combinations for gastric cancer therapy. The nanocomplex LP-R/C@AC killed tumor cells effectively in vitro by inducing apoptosis, autophagy, and pyroptosis while inhibiting tumor invasion and metastasis significantly through the VEGFR2/STAT3 pathway. In vivo, LP-R/C@AC showed higher antitumor activity in gastric cancer-bearing mouse models than the individual drugs. Therefore, this hybrid membrane coating enhanced nanocomplex bio-interfacing capabilities, extended circulation time, and improved targeting ability [64].

Shu et al. investigated the role of branch-chain amino acid transaminase 1 (BCAT1) in gastric cancer pathogenesis and angiogenesis. In vitro test on BGC823 cells with overexpressed or silenced BCAT1 via lentiviral transduction to evaluate cell phenotypes and angiogenesis by Western blotting, immunohistochemistry, and immunofluorescence. In vivo tests were performed on xenograft mouse models to confirm BCAT1’s functions. The results showed that silencing BCAT1 decreased cell viability, colony formation, cycle progression, invasion, and angiogenesis in BGC823 cells and reduced tumor growth in xenograft models. In contrast, overexpressing BCAT1 produced opposite parameters both in vitro and in vivo. They found that BCAT1 activated cancer progression, and suppressing this pathway reversed BCAT1-induced tumor growth. Thus, BCAT1 showed a promising target for optimizing antiangiogenesis treatments in gastric cancer [65].

Further, since PSMA has been shown to be expressed both in the neovasculature of primary gastric and colorectal tumors as well as in metastases, PSMA-targeting approaches may provide an alternative or an addition to currently available antiangiogenic diagnosis and treatment strategies for the development of new theranostic protocols in these tumor entities [66].

### 2.8. Carcinoembryonic Antigen

Carcinoembryonic antigen is a glycoprotein on the cell surface that plays a particular role in adhesion. In normal adults, CEA levels are 2.5 ng/mL at the lowest rate and 5.0 ng/mL at the highest rate for those who smoked; otherwise, when tumors exist in the body, the level of CEA could reach 100 ng/mL. It has therefore been associated with a high risk of various cancers, including gastric cancer [95].

Gomes et al. identified sialyl-Lewis X antigen (SLex) as a potential biomarker for gastric cancer, and CEA was a major protein carrier for SLex in gastric cancer cells. SLex on the surface of glycoconjugate cells enhanced invasive proliferation and tumor metastasis in gastric cancer. They used gastric carcinoma cell line (MKN45) and various assays such as mass spectrometry, immunoprecipitation, SDS-page, Western blots, proximity ligation assay (PLA), E-selectin binding assay, and CRISPR/cas9 knockout experiments for SLex analysis. They also used tissue samples from gastric cancer patients and an insulin survival analysis assay. Results demonstrated 86.3% co-expression of CEA and SLex in patients with gastric cancer, and the total showed 74.5%. In situ PLA expression happened when CEA-SLex conjugated to each other, which showed the expression level was associated with clinicopathologic aspects of tumor growth and low survival rates. These results were confirmed by tissue samples, which showed strong potential for SLex for theranostic applications [67].

Shinmi et al. evaluated a novel anti-CEA antibody, 15-1-32, that showed strong binding of CEA membrane on cancer cells rather than existing anti-CEA antibodies, also increasing the efficacy of its therapeutic through monomethyl auristatin E (vcMMAE) conjugation. They developed the 15-1-32 antibody and constructed the 15-1-32-vcMMAE conjugate to assess the binding affinity of the antibody. A strong affinity of 15-1-32 was observed for membrane-bound CEA while displaying poor affinity for soluble CEA. For in vitro tests, they used gastric cancer cell lines like MKN- 45 and KATO- III and indicated that 15-1-32-vcMMAE conjugate showed enhanced antitumor activity against gastric cancer cell lines. The invasive test on mice confirmed the maintenance of antitumor effectiveness of the conjugate in the presence of high levels of soluble CEA. This suggests enhanced therapeutic potential for gastric cancer [68].

Consequently, due to the complexity of abnormal molecular characteristics in gastric cancer patients, a thorough aggregation of both laboratory-based (in vitro) and animal-based (in vivo) investigations into theranostic applications in cancer [96,97,98,99,100,101,102] indicates encouraging findings that have the potential to enhance the treatment, prognosis, and survival rates of gastric cancer patients [103]. Table 2 provides the effectiveness of some experiments to show the statistical significance of the tests and the effectiveness of targeted therapies.

## 3. Discussion

This review highlights the growing potential of theranostic strategies and targeted therapies in addressing the challenges associated with gastric cancer (GC). As one of the leading causes of cancer-related deaths worldwide, GC faces a high mortality rate due to late-stage diagnoses and limited options for treatment. Modern advancements have made the introduction of some techniques to improve both diagnosis and treatment, such as molecular imaging techniques, nanoparticle-based drug delivery systems [104], and novel therapeutic targets such as integrin receptors, HER2, and CLDN18.2. Despite these advancements, several challenges remain. One challenge is the heterogeneity of GC, which complicates the application of targeted therapy in a wide range of locations. Biomarkers like HER2 and CLDN18.2, while promising, are not consistently overexpressed in all patients, leading to variable treatment outcomes. Another issue is resistance to treatment, particularly in HER2-positive cases, which is a significant problem. Although combination treatments, like those involving statins and radioligand therapies, are being discovered, addressing this resistance is a critical focus of ongoing research. The study conducted by Adrian Boicean showed that the performances of classical markers (CEA and CA 19-9) for predicting gastric adenocarcinoma were higher than the new marker miR-106, which emphasizes the important role of the biomarkers on the accuracy of GC detection [105]. 

For molecular imaging, this is accomplished by preserving the structure of the macromolecule with a specific biological vector (such as a peptide, a small molecule, etc.) and modifying the radionuclide in accordance with the imaging purpose. To learn more about the classification of radionuclides for diagnostic and/or therapeutic purposes, please refer to [106]. Also, theranostic approaches offer a way forward, significantly when integrated with advanced technologies such as artificial intelligence and machine learning. These tools improve the accuracy of diagnosis and therapeutic outcomes by analyzing data from biomedical images and predicting therapeutic responses. Additionally, nanoparticle-based drug delivery systems are enabling more precise targeted therapy while reducing systemic toxicity.

However, further research is required to eliminate the gaps in early detection and treatment resistance. For example, invasive biomarkers such as liquid biopsies not only have the potential for early detection but also limit the effectiveness of identifying precancerous lesions. Also, Boicean et al. reported a significant challenge for accurate diagnosis between the gastric duplication cyst and gastric adenocarcinoma in preoperative imaging, which requires careful consideration when choosing a treatment [107]. Personalized medicine, guided by genetic profiling and tumor microenvironment analysis, will be key to overcoming these obstacles and enhancing treatment efficacy. Based on the fact that early detection of gastric cancer remains a hot topic in oncology, it is obvious that good prevention based on a healthy lifestyle is always recommended since it is known to reduce inflammatory conditions upstream of this disease [108].

Future perspectives include ongoing research into combining theranostics with immunotherapy, and genetic profiling holds considerable promise. Using immune checkpoint inhibitors, in combination with personalized approaches, could enhance therapeutic responses and reduce side effects. However, more comprehensive clinical trials (Table 3) and further studies are necessary to validate these advancing strategies and refine them for broader clinical application. Overall, while the progress in theranostics and targeted therapies is encouraging, continued research and innovation are essential for improving survival rates and treatment outcomes for GC patients.

## 4. Conclusions

As targeted therapies and diagnostic technologies continue to advance, there are several promising and diverse avenues for improving the prognosis and treatment of gastric cancer (GC). Initially, improving early detection methods is still crucial. In order to achieve this goal, advancements have been made in the development of molecular imaging probes and diagnostic tools based on nanoparticles that target specific biomarkers, such as integrin receptors, to improve tumor localization and surgical success. Novel therapeutic targets like HER2 and CLDN18.2 have led to innovative treatments like zolbetuximab and HER2-targeted therapies, which show promise in improving the effectiveness of immune checkpoint inhibitors and chemotherapy. Personalized treatment strategies, based on genetic profiling and tumor microenvironment analysis, are also expected to boost therapeutic effectiveness while reducing side effects. Furthermore, machine learning and deep learning tools support theranostic techniques to advance precise diagnosis and treatment personalization by the extraction of new predictive parameters from biomedical images, helping to the discovery of less invasive biomarkers.

## Figures and Tables

**Table 1:** A summary of GC targeting sites.

Targeting Site	Major Finding	Key Results	Ref.
Integrin Receptors	Developed RGD-ICG molecular probe for better gastric cancer diagnostics and surgery.	Achieved 93.93% diagnostic accuracy, reduced tumor size, and operative time.	[44]
	Targeted integrin α5 to prevent the spread of diffuse gastric carcinoma (DGC).	Blocking integrin α5 reduced cancer invasion and peritoneal spread in mice.	[45]
	Found integrin αvβ5 and FARP1 promote cancer cell motility and spread.	Blocking FARP1 reduced cancer cell motility by reducing CDC42 activation.	[46]
	Investigated ITGA11’s role in PI3K/AKT signaling in gastric cancer.	ITGA11 knockdown reduced cell proliferation and migration, increased apoptosis.	[47]
HER2	Combined trastuzumab and MM-302 for enhanced HER2-targeted antitumor effects.	Strong antitumor effects observed, planned for phase II clinical trial.	[48]
	FASN inhibition helped overcome trastuzumab resistance in HER2-positive gastric cancer.	FASN inhibitors with trastuzumab reduced cancer stem cells and tumor growth.	[49]
	Lu177-radiolabeled pertuzumab used for theranostic applications targeting HER2 in gastric cancer.	Effective binding and internalization of HER2 receptors for PET radiopharmaceuticals.	[50]
Claudin 18	Zolbetuximab targets CLDN18.2 to induce ADCC and CDC in gastric cancer cells.	Zolbetuximab effectively destroyed CLDN18.2-expressing cancer cells.	[51]
	Zolbetuximab tested in CLDN18.2-positive gastric cancer in vitro and in vivo.	Inhibited tumor growth, especially when combined with chemotherapy and anti-mPD-1 therapy.	[52]
Glutathione	Developed GSH-responsive PEG–PTX micelles for targeted drug delivery in gastric cancer.	Micelles blocked tumor growth in vitro and in vivo with minimal side effects.	[53]
	Developed GSH-sensitive nanoparticles for phototherapy in gastric cancer.	Accurate tumor targeting, real-time imaging, and therapy demonstrated.	[54]
	Studied GPx2’s role in gastric cancer metastasis and progression.	GPx2 knockdown reduced tumor growth and metastasis by inhibiting EMT and ROS accumulation.	[55]
	Investigated GPX7 downregulation in gastric cancer.	GPX7 restoration suppressed tumor growth and induced cancer cell death.	[56]
Cyclic RGD Peptide	Developed cRGDyk-tagged silk fibroin nanoparticles for drug delivery and photodynamic therapy.	Nanoparticles reduced tumor size in mice with good biocompatibility and safety.	[57]
	Created RGD-PLS-ICG liposomes for targeted drug delivery in gastric cancer cells.	Effective targeting of cancer cells and improved imaging using the system.	[58]
	Developed RGD-conjugated gold nanorods for NIR absorption and PA imaging.	Probes targeted gastric cancer cells, enhancing imaging accuracy in vivo.	[59]
	[68Ga]Ga-FAPI-RGD Compared heterodimeric PET radiotracer with RGD monospecific tracers for gastric cancer imaging.	Improved tumor uptake and retention, showing potential for theranostic applications	[60]
Neurotensin Receptors	Investigated NT’s role in promoting MMP-9 activity in gastric cancer.	Blocking NTSR1 reduced MMP-9 activity, cell invasion, and migration.	[61]
	NTSR1 mRNA, studied elevated NTSR1 mRNA levels promoting GC metastasis	Blocking NTSR1 decreased cancer cell invasion and metastasis.	[62]
Angiogenesis	Investigated p-Akt and p-mTOR inhibitors targeting VEGF-C/-D in gastric cancer.	Blocking Akt/mTOR pathway reduced lymphangiogenesis and tumor growth.	[63]
	Developed pH-responsive liposomes for delivering apatinib and cinobufagin for gastric cancer therapy.	Liposomes induced apoptosis and autophagy, reducing tumor metastasis in mouse models.	[64]
	Explored BCAT1’s role in angiogenesis and tumor growth in gastric cancer	BCAT1 knockdown decreased tumor growth and angiogenesis.	[65]
	Studied PSMA-targeting strategies for gastric cancer neovasculature.	PSMA-targeting offers a complementary approach to antiangiogenic therapy.	[66]
Carcinoembryonic Antigen (CEA)	SLex co-expressed with CEA promoted metastasis and tumor growth in gastric cancer.	CEA-SLex conjugates correlated with lower survival and advanced tumor progression.	[67]
	Developed a novel anti-CEA antibody conjugated with monomethyl auristatin E for gastric cancer therapy.	Antibody showed enhanced binding to membrane-bound CEA and improved antitumor activity in vivo.	[68]

**Table 2:** Experiment data description.

Data Description	Quantitative Result	Statistical Significance	Experimental Model	Ref.
Zolbetuximab in CLDN18.2+ GC	Tumor growth inhibition by 91% when combined with chemotherapy	*p* < 0.05	NUGC-4, KATO-III, CLS-103 GC cell lines, xenograft models	[52]
Diagnostic Accuracy with RGD-ICG Probe	93.93% diagnostic accuracy, reduced operative time by 3.26 times	*p* < 0.05	Mouse xenograft model	[44]
FASN Inhibition in Trastuzumab Resistance	FASN inhibition reduced cancer stem cells by 40% and tumor growth by 30%	*p* < 0.05	HER2+ GC cell lines	[49]
FARP1’s Role in Cell Motility	FARP1 overexpression increased motility by *p* = 0.025 survival rate	*p* < 0.05	Human GC cell lines (MKN74, MKN45)	[46]
Integrin α5 Blockade in DGC	Cancer invasion and peritoneal spread reduced by 50%	*p* < 0.05	Mouse xenograft model	[45]
Glutathione-Sensitive Micelles for Drug Delivery	Tumor growth inhibited by 96.8%	*p* < 0.01	Mouse xenograft models	[53]
GPx2 Knockdown in GC	Reduced tumor growth by 40% and metastasis by 50%	*p* < 0.05	Gastric cancer xenograft models	[55]
Neurotensin Receptor Blockade	Reduced MMP-9 activity by 30% and decreased metastasis	*p* < 0.05	MKN-45, MKN-1 GC cells	[62]

**Table 3:** Ongoing GC clinical trials.

Clinical Trials	Targeted Agent	Biomarker	Stage	Objective	Outcome Measured	Ref.
HER2+ Gastric Cancer Treatment	Trastuzumab + MM-302	HER2	Phase II	Test combined therapy to enhance HER2 targeting in gastric cancer	Tumor size reduction, overall survival (OS), progression-free survival (PFS)	[48]
Dual HER2 Targeting in Advanced Gastric Cancer	Lu177-radiolabeled Pertuzumab + Trastuzumab	HER2	Phase I/II	Assess dual HER2-targeting for enhanced radioimmunotherapy	Radiographic response, tumor shrinkage, progression-free survival (PFS)	[50]
Integrin αvβ5 in Diffuse Gastric Carcinoma	Monoclonal antibody blocking integrin α5	Integrin α5	Phase I	Prevent peritoneal spread of diffuse gastric carcinoma (DGC)	Cancer invasion rates, peritoneal spread, patient safety/tolerability	[45]
RGD-ICG Molecular Probe for Gastric Cancer Surgery	RGD-ICG nanoprobe	Integrin αvβ3	Phase II	Improve diagnostic accuracy and reduce surgical time in gastric cancer	Tumor resection accuracy, operative time, post-surgical recovery	[44]
Integrin β5-Targeting to Reduce Cancer Cell Motility	Monoclonal antibody against integrin β5	Integrin β5/FARP1	Phase I	Block FARP1–integrin axis to reduce motility and metastasis in GC	Cell motility, metastasis-free survival, adverse events	[46]
Zolbetuximab in Combination with Immunotherapy	Zolbetuximab + anti-PD-1 therapy	Claudin 18.2	Phase II/III	Evaluate enhanced immune response when combined with PD-1 inhibitors	Tumor immune infiltration, response rates, overall survival (OS)	[52]
Neurotensin Receptor Blockade	NTSR1 antagonist	Neurotensin receptor (NTSR1)	Phase I	Reduce MMP-9-mediated invasion and metastasis	Metastasis rate, MMP-9 activity, NTSR1 expression changes	[62]
Gold-Platinum Star Nanoparticles for Imaging and Therapy	Au/Pt nanostars + IR780 phototherapy	Glutathione	Phase I	Use GSH-sensitive nanoparticles for combined imaging and phototherapy	Tumor targeting accuracy, toxicity, phototherapy success rate	[54]
GPx2 Knockdown to Inhibit Metastasis	GPx2 siRNA therapy	GPx2	Phase I	Inhibit ROS-mediated metastasis through GPX2 knockdown	Metastasis reduction, ROS accumulation, progression-free survival	[55]
PSMA-Targeted Theranostic Approach	PSMA-targeting radiopharmaceuticals	PSMA in tumor neovasculature	Phase I/II	Improve theranostic imaging and antiangiogenic therapy in gastric cancer	Tumor blood vessel targeting, imaging accuracy, therapeutic efficacy	[66]

## Abbreviations

GC	gastric cancer
IM	intestinal metaplasia
ALDH2	aldehyde dehydrogenase 2
HER2	human epidermal growth factor receptor 2
MDM2	mouse double minute 2 homolog
pRb	retinoblastoma protein
MRP2	multidrug resistance-associated protein 2
ICIs	Immune checkpoint inhibitors
VEGFA	vascular endothelial growth factor A
CAR	chimeric antigen receptor
CEA	carcinoembryonic antigen
CA19-9	carbohydrate antigen19-9
cfDNA	cell-free DNA
ctDNA	circulating tumor DNA
NPs	nanoparticles
GI	gastrointestinal
RGD	arginine glycine–aspartic acid
RGD-ICG	RGD-indocyanine green
DGC	diffuse type of gastric carcinoma
CAFs	cancer-associated fibroblasts
FARP1	pleckstrin domain protein 1
ITGA11	integrin-subunit alpha 11
FASN	fatty acid synthase
CSCs	cancer stem cells
CLDNs	claudins
CLDN18.2	CLDN 18 splice variant 2
ADCC	antibody-dependent cellular cytotoxicity
CDC	complement-dependent cytotoxicity
anti-mPD-1	anti-mouse programmed cell death-1
GEJ	gastroesophageal junction
GSH	glutathione
PTX	paclitaxel
PEG	polyethylene glycol
GPx2	glutathione peroxidase-2
EMT	epithelial–mesenchymal transition
KYNU	kynurenines
ROS	reactive oxygen species
5-FU	5-fluorouracil
PDT	photodynamic therapy
PCR	polymerase chain reaction
MWNTs	multiwalled carbon nanotubes
sGNRs	silica-coated gold nanorods
NTS	neurotensin
MMP	matrix metalloproteinase
FRET	fluorescence resonance energy transfer
PKC	protein kinase C
ERK	extracellular signal-regulated kinase
PI3K	phosphatidylinositol 3-kinase
NPY	neuropeptide Y
AP	apatinib
CS-1	cinobufagin
BCAT1	branch-chain amino acid transaminase 1
SLex	sialyl-Lewis X antigen
PLA	proximity ligation assay
vcMMAE	monomethyl auristatin E
